# The hidden peril: Ecuador's struggle with substandard and falsified medicines

**DOI:** 10.3389/fpubh.2026.1773488

**Published:** 2026-02-04

**Authors:** Jorge Vasconez-Gonzalez, Juan S. Izquierdo-Condoy, Esteban Ortiz-Prado

**Affiliations:** One Health Research Group, Universidad de Las Américas, Quito, Ecuador

**Keywords:** substandard medicines, falsified medicines, illicit pharmaceutical trade, drug supply chain traceability, antimicrobial resistance

## 1 Introduction

One of the key pillars for ensuring the health and wellbeing of populations is access to medicines and vaccines that are safe, effective, and affordable (1). Although numerous structures and institutions have been established worldwide to regulate the production, sale, and distribution of medicines, an unregulated market persists in many low- and middle-income countries. This includes improvised points of sale at local fairs and street markets where pharmaceuticals can be purchased (1, 2). In these settings, the prevalence of falsified and substandard medicines poses a substantial public health threat. It exposes patients to the harmful effects of undeclared or unauthorized substances, excipients, and contaminants (3, 4).

In Latin America, this market has expanded considerably. It is estimated that roughly 30% of medicines sold in the region are counterfeit (5). According to the 2023 Global Organized Crime Index, countries with the highest rates of product falsification (including a strong emphasis on medicines) include Paraguay and Peru (index 9), followed by Mexico (8.5), Colombia (7.5), and Brazil (7) (6). In their study, Rojas-Cortés et al. reported that, in Latin America, the most affected medicines were anti-infectives (16.7%), analgesics and palliative-care medicines (14.2%), and hormones, other endocrine medicines, and contraceptives (9.5%) (4).

In this document, we adopt the World Health Organization (WHO) taxonomy of substandard and falsified (SF) medical products: substandard products fail to meet quality specifications without implying intent to deceive, whereas falsified products involve fraudulent misrepresentation of identity, composition, or origin (7). In Ecuador, the term “irregular medicines” is frequently used as a broad category. Here, we use it to encompass unregistered/unauthorized products, expired or improperly stored products, adulterated products (containing undeclared substances), smuggled products, and medical samples or institutional-use products inappropriately commercialized, in addition to SF products.

## 2 The Ecuadorian context: a public health crisis

Ecuador is currently facing a public health crisis, including widespread medicine shortages. These have been highlighted by 38 patient organizations representing complex conditions, united under the National Health Alliance. The Ecuadorian Social Security Institute (IESS) has declared an emergency in pharmaceutical procurement to tackle these deficits (8, 9). Despite the oversight role of the National Agency for Regulation, Control, and Health Surveillance (ARCSA) which handles regulation, technical control, and surveillance of medicines, nutraceuticals, biological products, processed natural products for medicinal use, homeopathic medicines, and dental products illicit pharmaceutical sales continue to escalate (10).

In 2025, ARCSA conducted nationwide operations, inspecting 595 pharmaceutical establishments near 200 hospitals. Authorities seized 32,503 irregular medicines, dietary supplements, and homeopathic products (11). Quito was the hardest hit, with 20,482 irregular items identified. Key hotspots included:

- Surroundings of the Carlos Andrade Marín Hospital (IESS): Over 9,800 irregular products detected.
- Vicinity of the Military Hospital: More than 5,000 medical samples sold illegally.
- Area around the Luz Elena Arismendi Gyneco-Obstetric Hospital (MSP): 1,767 expired medicines and devices confiscated (11, 12)

On the other hand, in the city of Guayaquil on Ecuador's coast, a clandestine pharmacy was discovered containing more than 1,800 medicines, including expired and prohibited products. Additionally, several street stalls and tents were identified, offering more than 470 additional products (13). In addition, in front of an MSP hospital, 1,300 medicines were seized, including fentanyl and expired products. (14). Meanwhile, in other areas, 7,981 products were confiscated, including counterfeit, institutional-use, and smuggled medicines (15).

This illicit trade is not new in Ecuador. Past incidents include a 2016 seizure of antibiotics, anti-inflammatories, antiepileptics, antihypertensives, and other medicines for labeling violations and lack of sanitary registration (16). In 2017, tons of suspected counterfeit medicines were confiscated in Azuay, El Oro, Morona Santiago, Loja, and Cañar provinces. Known as the “Mediveza case,” this involved 18 tons of counterfeit drugs—the largest seizure in Ecuador's history (17).

## 3 Public health implications

These types of products lack guarantees that ensure their quality, authenticity, and proper storage. Therefore, their use exposes patients to a series of risks, including poisoning, worsening of their health due to inappropriate treatments, and they can even lead to death (7, 18). Another issue to highlight is that, on many occasions, they contain low-quality ingredients or active ingredients, or in inadequate amounts (insufficient quantities), which can lead to therapeutic failure and even contribute to the development of drug-resistant pathogens, thereby promoting the spread of resistant (7, 19). On the other hand, it is important to highlight that, in addition to health damages, these irregularities can generate a significant economic impact due to the billions of dollars lost annually from wasted treatments, increased healthcare costs, and decreased productivity (7).

## 4 Policy recommendations

To address this problem, it is necessary to urgently implement a strategy that combines both process controls, such as electronic prescriptions and serialization, along with contextual controls, including audits and public reporting channels, as well as input controls, such as transparent product procurement and robust logistics. Short-term strategies with immediate impact should focus on audits, verification systems, citizen reporting channels, and the management of expired products. On the other hand, long-term strategies should promote reforms such as serialization and electronic prescription, in order to efficiently and optimally establish complete traceability from the acquisition of medicines to their dispensation to patients. Critical to success are data interoperability, transparent public metrics, and seamless collaboration across agencies (ARCSA, MSP/IESS, police, and the Prosecutor's Office); otherwise, sporadic actions will only shift the problem elsewhere (Table 1).

**Table 1:** Integrated policy package to curb irregular medicines in Ecuador.

**Measure**	**Key actions (what it entails)**	**Public health objective**	**Monitoring indicators (KPIs)**	**Timeframe**	**Key actors**
Serialization and traceability (GS1, track-and-trace)	Unit-level GS1/Datamatrix coding, step-wise aggregation, verification at dispensing, interoperability with ARCSA	Prevent entry of SF/smuggled products and ensure supply-chain visibility	% units serialized; % dispensings verified; lot/serial alerts; time-to-recall	Medium	ARCSA, MSP/IESS, manufacturers and distributors, pharmacies, GS1
E-prescription and control of narcotics/opioids	National e-prescribing platform; identity validation; limits and audit trails for fentanyl/analogs; linkage to surveillance	Reduce diversion, inappropriate prescribing, and illicit sales	% electronic prescriptions; detected discrepancies; cancellation rate; alerts for atypical prescribing	Short–Medium	MSP/IESS, hospitals and pharmacies, professional colleges, ARCSA
Transparent public procurement	Framework contracts; public stock dashboards; inventory APIs; anti-corruption and performance clauses	Prevent stock-outs and risky purchases	Fill rate; stock-out days per item; replenishment lead time; % contracts with performance targets	Short	MSP/IESS, SERCOP/finance, Comptroller General, hospitals
Citizen verification and complaint channels	ARCSA app/web to verify registrations/serials (QR); public education campaigns; hotline for complaints	Empower users and accelerate detection	App downloads/queries; complaints received; response time; % cases sanctioned	Short	ARCSA, MSP, civil society, media
Targeted audits in high-risk zones	Data-driven inspections around hospitals and commercial corridors; joint operations with Prosecutor's Office/Police	Dismantle street/clandestine sales	Number of operations; items seized; recidivism rate; effective closures	Short	ARCSA, Police, Prosecutor's Office, municipalities
Safe management of expired products (reverse logistics)	Collection points; certified transport and destruction; agreements with waste managers	Prevent re-entry of expired products into the market	Tons collected; % facilities enrolled; destruction audits	Short	MSP/ARCSA, municipalities, environmental managers, pharmacies

## 5 Conclusion

The ongoing illicit trade in medicines—encompassing falsified, substandard, unregistered, adulterated, expired, and prohibited items like medical samples or institutional-use products poses a profound public health threat in Ecuador. To mitigate this, it is essential to bolster regulatory oversight of procurement and sales, raise patient awareness of associated risks and verification methods, and guarantee equitable access to safe, effective, and affordable therapies. These steps are crucial to deter reliance on hazardous alternatives and safeguard community health.
